# The influence of physical exercise on college students’ life satisfaction: The chain mediating role of self-control and psychological distress

**DOI:** 10.3389/fpsyg.2023.1071615

**Published:** 2023-01-24

**Authors:** Guang-Yu Zhou, Bin Yang, Hao Li, Quan-Sheng Feng, Wan-Yi Chen

**Affiliations:** Department of Physical, College of Education and Sports Sciences, Jingzhou, China

**Keywords:** physical exercise, life satisfaction, self-control, psychological distress, undergraduate

## Abstract

**Objective:**

To explore the relationship between physical exercise and life satisfaction among college students and test the dual mediating role of self-control and psychological distress between them.

**Methods:**

A sample of 526 Chinese college students completed questionnaires regarding physical exercise, life satisfaction, self-control and psychological distress, of which 38.4% were boys.

**Results:**

Path analyzes indicated that physical exercise was positively correlated with life satisfaction, and this link could be mediated by self-control and psychological distress.

**Conclusion:**

The present study identifies the potential underlying mechanism by which physical exercise is associated with the life satisfaction of college students, which has important implications for theory and prevention.

## 1. Introduction

Life satisfaction is an individual’s overall evaluation of his or her nearest living environment and living conditions (Shin and Johnson, 1978) and is an individual’s overall evaluation of the environment (Teodora et al., 2021). An individual’s positive satisfaction with their life as a whole or with specific components of their life plays a key role in achieving their life goals (Teodora et al., 2021). At the freshman stage of university, students are in a special period of cognitive development. Students’ evaluation of their living situation is an important assessment indicator of their well-being, and good life satisfaction can help them to enhance their studies and promote physical and mental monitoring of their development (Hui et al., 2021). Therefore, it is of positive significance to study the level of life satisfaction and the factors influencing it in a group of Chinese university students.

Previous studies have shown that the level of personal life satisfaction is not only influenced by the individual’s internal emotions (such as anger, frustration), but also influenced by external social factors (such as work environment) and behavioral lifestyles (such as physical and cultural activities) (Cruz-Ferreira et al., 2011). Among these influences, people’s behavior and lifestyles are of wide concern to society (Arslan and Akkas, 2014; Liu et al., 2021). Physical exercise is a physical activity that aims to develop physical fitness, improve physical and mental health and enrich the diversity of one’s life through a variety of reasonable physical means according to one’s needs (Han, 2015). Physical exercise strengthens an individual’s immune system, maintains vigor and reduces the incidence of disease (Yang and Liang, 2011). At the same time, physical exercise can also relieve negative heart emotions such as tension and anxiety caused by negative life events and help them develop a positive and optimistic attitude toward life (Cao et al., 2021). During exercise, the brain secretes glucagon, which stimulates the hypothalamus to produce a pleasurable emotional experience. Therefore, physical activity will alleviate individuals’ negative emotions and help them develop their mental health in a positive direction (Cao et al., 2021).

### 1.1. The association between physical exercise and life satisfaction

Data from previous studies point to a strong link between physical activity and life satisfaction among university students (Liu et al., 2021). In recent years, there has been an increase in research on the relationship between physical activity and life satisfaction, and the positive effects of physical activity on life satisfaction are gaining attention from researchers (Chen, 2005). Mehmet and Fatih (2018) proposed that regular physical activity actively increases personal satisfaction with life, Jetzke and Mutz (2020) physical activity was found to have an important role in improving the well-being of young people. At the same time, studies have shown that university students who participate in sports show higher levels of satisfaction with themselves than their peers who do not participate in sports (Arslan and Akkas, 2014). Therefore, it is relevant to study the issue of the effect of physical activity on life satisfaction among university students from a physical and mental perspective. Based on the research, this study inferred that physical activity significantly and positively predicted life satisfaction among university students.

### 1.2. Self-control as a mediator

Research has found that people with high self-control tend to have better social adjustment skills (such as interpersonal skills and emotional regulation), which leads to better performance in their daily studies or work and therefore higher subjective well-being (Tan and Guo, 2008). Life satisfaction is considered to be an important cognitive component of subjective well-being (Diener, 1996). Self-control is a process by which individuals wish to improve their original behavior and thinking by reducing their own undesirable desires, a process by which new ways of behaving and thinking replace old ways of behaving and thinking (Tan and Guo, 2008). On the other hand, it has been found in self-control studies that self-control can be induced through physical exercise (Zhang et al., 2018). Baumeister et al. (2007) proposes in the self-control model theory that ‘self-control as an energy resource is depleted by the continuous performance of self-control tasks’. Therefore, if university students are able to control their behavior or thoughts in the process of participating in physical exercise, they will be able to improve their level of self-control in the process of completing physical exercise within the prescribed time (Cheng and Wang, 2020). Based on the studies, this study infers that self-control plays a mediating role in the effect of physical activity on college students’ life satisfaction.

### 1.3. Psychological distress as a mediator

Psychological distress, also known as psychological distress, is an unpleasant emotional experience caused by a variety of factors, including psychological, social and spiritual dimensions, and can develop from the common fear, sadness, and vulnerability to anxiety, depression, and fear (Hamer et al., 2009). Research shows that physical activity is a significant negative predictor of psychological distress (Sun and Dai, 2007). For college students, active participation in physical activity is beneficial in regulating mood, enhancing sensory perception and enhancing internal motivation (McMahon et al., 2017). Psychological measures were administered to subjects after participation in exercise and the data showed a significant decrease in both depressive states, anxiety and stress or psychological disorders (Best, 2010). Individuals who do not participate in physical activity will experience anxiety and depression over a longer period of time than those who are physically active and regular (Sun and Dai, 2007). The study also found that individuals with low levels of psychological distress had high levels of life satisfaction (Yennurajalingam et al., 2018). Happiness is an essential characteristic of mental health, and when individuals have fewer negative emotions, they achieve a higher level of subjective well-being (Yu, 2022), in other words, have better life satisfaction. Based on the findings, this study infers that psychological distress mediates the effect of physical activity on life satisfaction among university students.

### 1.4. The chain mediating effect of self-control and psychological distress

Self-control has been studied as an important component of mental health (Duckworth et al., 2019). Self-controlled, temptation-denying decision-making provides clear positive self-signals (Dhar and Wertenbroch, 2012), and self-control within an individual is navigated from different aspects using various elements of psychology (Ge and Ho, 2021). Research has shown that self-control has two effects on mental states, one that promotes positive mental states (such as efficacy, self-esteem and life satisfaction) and one that inhibits negative mental states (such as anxiety, stress and suicidal ideation) (Qi and Zhang, 2014). Research shows that self-control inhibits negative mental states more than it facilitates positive mental states (de Ridder et al., 2012). And, individuals with high self-control have lower levels of psychological distress (Ge et al., 2021; Shi and Ma, 2021). Therefore, this study infers that self-control and psychological distress play a chain mediating role in the effect of physical activity on life satisfaction among university students.

### 1.5. The present study

Although it has been suggested that self-control and psychological distress are associated with physical activity and life satisfaction among university students, it remains unclear how self-control and psychological distress affect this relationship. To our knowledge, this is the first study to consider the mediating effects of self-control and psychological distress. The present study contributes to a better understanding of the relationship between physical activity and life satisfaction among university students and contributes to the generalization of social control theory and general stress theory in the field of life satisfaction.

In many schools in China, at the end of the semester, in order for students to excel in exams, teachers of specialist subjects often take time away from PE lessons for students to revise. Teachers of specialist subjects believe that less PE lessons are irrelevant to their students. The purpose of this study is to raise awareness of the importance of physical exercise, to change the past disdain for physical exercise and physical education classes, to make society more aware of students’ psychological problems and to have practical implications for the reform and development of the education sector.

In short, using Chinese college students as participants, this study tested the mediating effect of self-control and psychological distress on the relationship between physical exercise and life satisfaction. Based on previous empirical research, we put forward four hypotheses: (1) physical exercise may be positively correlated with life satisfaction; (2) self-control may be the intermediary between physical exercise and life satisfaction; (3) psychological distress may be the intermediary between physical exercise and life satisfaction and (4) self-control and psychological distress play a chain mediating role between physical exercise and life satisfaction.

## 2. Methods

### 2.1. Participants

The participants in this study were recruited from two middle schools in Hubei, China through random cluster sampling. A total of 600 questionnaires were distributed, of which 526 were valid, and the effective recovery rate was 87.67%. There were 202 boys and 324 girls, with an average age of 18.82 (range = 17–19; SD = 1.706).

### 2.2. Procedures

The present study was approved by the Research Ethics Committee of the College of Education and Sports Sciences, Yangtze University. Convenience sampling was adopted to choose six to seven classes in each grade. Participants and their parents or legal guardians were provided with written consent forms, which informed them that personal information would be kept confidential and their responses would be used only for research purposes. The data were collected by trained senior students majoring in psychology during class time. To encourage honest reporting, college students were given approximately 30 min to complete the anonymous questionnaires.

### 2.3. Measures

#### 2.3.1. Physical exercise

The physical training of college students was assessed using the “Physical Activity Rating Scale” developed by Liang (1994). The scale assesses the physical exercise activity level from three aspects: exercise intensity, exercise time and exercise frequency, each of which is divided into five levels, scored from 1 to 5. Calculated as: amount of physical activity = exercise intensity × (exercise time-1) × exercise frequency; maximum score 100 points, minimum score 0 points and higher score means higher individual exercise. The physical activity level can be divided into 3 levels by the conversion of scores, and the classification criteria are as follows: small exercise ≤19 points, 20 points ≤ moderate exercise ≤42 points and large exercise ≥43 points. In order to facilitate the classification of sports activity levels, according to previous studies, the original ≤19 small exercise levels were divided into two criteria: no exercise ≤4 points and 5 points ≤ small exercise ≤19 points, so the sports activity levels in this study were divided into four levels (Xia et al., 2018). Cronbach’s alpha coefficient for this questionnaire in this study was 0.60.

#### 2.3.2. Life satisfaction

Observed using the Life Satisfaction Scale used by Pavot and Diener (1993). There are 5 questions in this scale, which are scored by 7 points (“Strongly disagree” to “Strongly agree”). The level of life satisfaction was determined by the cumulative score of the questions. Cronbach’s alpha coefficient for this questionnaire in this study was 0.86.

#### 2.3.3. Self-control

Tan used the “self-control scale” for measurement (Tan and Guo, 2008). The scale consists of 19 topics, including five dimensions: individual impulse control level, lifestyle habits, resistance to temptation, focus on work, or study and rational entertainment. The scale is scored on a 5-point scale (“not at all” to “very much”). They are reverse scored except questions 1, 5, 11, and 14. The higher the score, the stronger the self-control ability. Cronbach’s alpha coefficient for this questionnaire in this study was 0.88.

#### 2.3.4. Psychological distress

The simplified Chinese version of the Depression Anxiety Stress Scale revised by Gong et al. (2010) was used, which consists of 21 questions and contains three dimensions: depression, stress, and anxiety. A 4-point score (“not met” to “very met”) was used, and the higher the score, the stronger the degree of psychological distress. The Cronbach’s alpha coefficient for this questionnaire in this study was 0.95.

### 2.4. Statistical analysis

Statistical analysis was performed using SPSS 24. Common method deviance testing was performed first, followed by descriptive statistical analysis and Pearson’s correlational analysis of physical exercise, life satisfaction, self-control, and psychological distress. And finally, we employed the SPSS macro PROCESS (model 6) suggested by Hayes to test the proposed mediation model (Hayes, 2013). This SPSS macro has been used to test mediating models in several studies, in which this SPSS macro showed higher statistical testability (Lian et al., 2018, 2021).

### 2.5. Common method deviation test

Exploratory factor analysis was used to test common method deviations (Zhou and You, 2004). Taking all the questions of each questionnaire as the items for exploratory factor analysis, the explanation rate of the first common factor precipitated is 28.87%, which is much less than the critical criterion of 40%, so there is no serious common method deviation in the data of this study.

## 3. Result

### 3.1. Descriptive statistics and associated analyzes

The results of correlation analysis of each variable (see Table 1) showed that physical exercise was significantly positively correlated with life satisfaction and self-control, and significantly negatively correlated with psychological distress; life satisfaction was significantly positively correlated with self-control and significantly negatively correlated with psychological distress and psychological distress was significantly negatively correlated with self-control.

**Table 1:** Descriptive statistics and associated analysis results.

	M	SD	1	2	3	4
1. Physical exercise	2.30	0.93				
2. Life satisfaction	3.98	1.41	0.09*			
3. Self-control	3.30	0.54	0.20**	0.43**		
4. Psychological distress	0.78	0.54	−0.14**	−0.40**	−0.52**	

*p < 0.05, **p < 0.01.

### 3.2. Mediation model testing analysis

Bootstrap (Fang et al., 2012) was used to analyze the mediating effect of self-control and psychological distress. After controlling for gender, age and grade, 5,000 samples were randomly selected to estimate the 95% confidence interval of the mediation effect to analyze the mediation effect of self-control and psychological distress in the effect of physical exercise on life satisfaction, and the analysis results are shown in Table 2.

**Table 2:** Regression analysis of relationships among variables.

Regression equation		Global fit metrics			Regression coefficient significance	
Outcome variables	Predictor variables	*R*	*R* ^2^	*F*	Beta	*T*
Life satisfaction	Gender	0.10	0.01	1.45	0.07	0.70
	Age				−0.02	−0.57
	Grade				−0.01	−0.12
	Physical exercise				0.10	2.20*
Self-control	Gender	0.24	0.06	8.04	0.01	0.06
	Age				−0.08	−1.85
	Grade				0.00	0.06
	Physical exercise				0.21	4.83***
Psychological distress	Gender	0.54	0.29	42.02	−0.14	−3.37**
	Age				−0.01	−0.20
	Grade				−0.00	−0.07
	Physical exercise				−0.07	−1.71
	Self-control				−0.52	−13.51***
Life satisfaction	Gender	0.48	0.23	25.75	−0.00	−0.03
	Age				0.01	0.19
	Grade				−0.01	−0.18
	Physical exercise				−0.01	−0.22
	Self-control				0.30	6.58***
	Psychological distress				−0.25	−5.41***

*p < 0.05, **p < 0.01, ***p < 0.001.

The overall effect of physical exercise on life satisfaction was significant (*β* = 0.10, *p* < 0.05), but the predictive effect on life satisfaction was no longer significant when the mediating variable was added (*β* = −0.01, *p* > 0.05). Physical exercise positively predicted self-control (*β* = 0.30, *p* < 0.001). Psychological distress negatively predicted life satisfaction (*β* = −0.25, *p* < 0.001).

**Figure 1:**
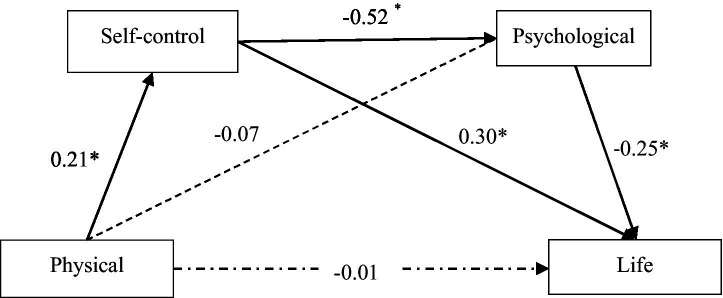
Schematic diagram of chain mediation. *P < 0.05.

Mediation effect analysis showed (see Figure 1; Table 3). Bootstrap 95% confidence intervals for the mediation effects of psychological distress and self-control did not include 0, indicating that psychological distress and self-control were mediating variables for physical exercise affecting life satisfaction. Among them, the mediating effect of self-control accounted for 60% of the total effect, and the chained mediating effect of self-control and psychological distress accounted for 30% of the total effect.

**Table 3:** Mediation effect test.

	Indirect effect value	Boot standard error	Boot CI lower	Boot CI upper	Relative mediation effect
Indirect effect 1	0.06	0.16	0.04	0.10	60%
Indirect effect 2	0.03	0.01	0.01	0.04	30%

## 4. Discussion

This study investigated the effect of physical exercise on life satisfaction in college students and the mechanisms of self-control and psychological distress. The results showed that physical exercise had no significant predictive effect on college students’ life satisfaction, but it could indirectly affect college students’ life satisfaction through the intermediary effect of self-control and the chain intermediary effect of self-control and psychological distress. In addition, in the regression analysis of psychological distress, it is also found that the degree of psychological distress in women is greater than that in men. This phenomenon may be due to the fact that women do not feel as secure as men in the university environment and therefore can possess more psychological distress (Etopio et al., 2019).

The direct effect of physical exercise on life satisfaction found in this study was not significant, consistent with the findings of McAuley et al. (2006) and Dai and Yao (2012). In McAuley et al. (2006) study, by constructing an empirical model of the relationship between physical exercise and individual life satisfaction, it was found that reducing the number of times an individual participates in physical exercise decreases an individual’s chances of having a successful experience in physical exercise, which leads to a reduction in self-efficacy. Subsequently, it is accompanied by a decline in health status, which ultimately affects people’s quality of life. Self-efficacy is closely linked to self-control (Chen et al., 2021). Therefore, physical exercise does not have a direct impact on life satisfaction as shown by previous findings but ultimately impacts life satisfaction through self-control and mental health status in turn.

This study further confirmed that self-control plays a completely mediating role between physical exercise and college students’ life satisfaction. On the one hand, college students’ physical exercise can significantly positively predict self-control. As an energy resource, self-control is continuously consumed in sports. If physical exercise is performed for a long time, this energy of self-control will be greatly increased in the body, so physical exercise will promote the improvement of self-control (Baumeister et al., 2007; Cheng and Wang, 2020). On the other hand, self-control significantly predicted college students’ life satisfaction. Self-control is one of the most powerful abilities of the human mind to benefit most from the human mind because people can adjust themselves to improve their fit with the self and environment, and when people feel happiest and healthiest is when they match perfectly (Tangney et al., 2004). People with high self-control adapt better to stress in life and work, and as a result, they have a higher sense of life satisfaction (Tangney et al., 2004; Tavis et al., 2007).

The intermediate link of the path “physical exercise–self-control–psychological distress–life satisfaction” is the chain intermediary composed of “self-control–psychological distress” and is an important bridge for physical exercise to affect college students’ life satisfaction. This suggests that in addition to self-control alone playing a mediating role between physical exercise and life satisfaction in college students, physical exercise can also affect psychological distress by affecting self-control and then play a role in life satisfaction. Through long-term effective physical exercise, individuals can improve their own self-control level (Zhang et al., 2018). In the process of physical exercise, with the individual requirements for self-movement and reasonable management of their own time, they can also improve their own self-control level. When individuals possess a high level of self-control, it will have a positive impact on their psychological state, thereby reducing the negative emotions of individuals and alleviating the adverse psychological state of the self (Yu, 2022). Therefore, individuals with a high level of self-control tend to produce less adverse psychological states, which, in turn, reduce the degree of psychological distress. When individuals have low levels of psychological distress, subjectively, people will have a positive attitude toward life, so individuals will obtain a good emotional experience and then obtain a high level of life satisfaction (Yu, 2022).

On the whole, this study explains the psychological mechanism of college students’ physical exercise affecting life satisfaction: physical exercise indirectly affects life satisfaction through independent mediation of self-control and chain mediation of self-control and psychological distress. Compared with previous studies, this study found that physical exercise does not directly affect individual life satisfaction, but indirectly affects individual life satisfaction through its effects on individual self-control and psychological distress. This study has important implications for improving college students’ life satisfaction. On the one hand, the educational administrators of colleges and universities should pay more attention to the daily physical exercise of college students, and at the same time, they should increase the publicity of the benefits of physical exercise to students. By strengthening the physical exercise of college students, they can reduce the negative psychological state of the students and improve their life satisfaction level. On the other hand, society and schools can pay more attention to the psychological state of college students and reduce the adverse events caused by their physical and mental conditions on campus or off campus.

## 5. Limitations and future directions

Limitations of this study and future directions should be noted. First, due to our cross-sectional research design, causality cannot be established. Future longitudinal or experimental studies can further examine the causal relationship between Physical Exercise and life Satisfaction. Second, self-reports may be subject to increased biases. Future studies should allow for a multidimensional approach to collect more objective. Finally, the results of the present study also need to be extended to a more representative sample of Chinese adolescents and to adolescents from other cultural backgrounds for a wider test.

## Data availability statement

The raw data supporting the conclusions of this article will be made available by the authors, without undue reservation.

## Ethics statement

The studies involving human participants were reviewed and approved by the Research Ethics Committee of the College of Education and Sports Sciences, Yangtze University. The patients/participants provided their written informed consent to participate in this study.

## Author contributions

G-YZ and W-YC designed the work. G-YZ collected the data. G-YZ and HL analyzed and drafted the manuscript. G-YZ, BY, HL, and Q-SF revised the manuscript. All authors contributed to the article and approved the submitted version.

## Conflict of interest

The authors declare that the research was conducted in the absence of any commercial or financial relationships that could be construed as a potential conflict of interest.

## Publisher’s note

All claims expressed in this article are solely those of the authors and do not necessarily represent those of their affiliated organizations, or those of the publisher, the editors and the reviewers. Any product that may be evaluated in this article, or claim that may be made by its manufacturer, is not guaranteed or endorsed by the publisher.
